# Supplementary material for: HSP90 as an evolutionary capacitor drives adaptive eye size reduction via atonal
Source: Nat Commun. 2025 Oct 20;16:9277. doi: 10.1038/s41467-025-65027-0 (PMC12537963; doi:10.1038/s41467-025-65027-0)
Supplement: Supplementary file 1 — Supplementary Information [file 41467_2025_65027_MOESM1_ESM.pdf]

Supplementary Information

HSP90 as an Evolutionary Capacitor Drives Adaptive Eye Size Reduction via Atonal

Rascha Sayed[1], Özge Şahin[1†], Mohammed Errbii[1†], Reshma R[1], Robert Peuß[2], Tobias Prüser[1], Lukas Schrader[1‡], Nora K. E. Schulz[1‡] and Joachim Kurtz[1]✉

[1]Institute for Evolution and Biodiversity, University of Münster, Hüfferstrasse 1, DE-48149 Münster, Germany. [2]Institute for Integrative Cell Biology and Physiology, University of Münster, Schlossplatz 8, DE-48143 Münster. [†]These authors contributed equally to this work. [‡]These authors contributed equally to this work.

✉email: joachim.kurtz@uni-muenster.de

Extended Data Figures

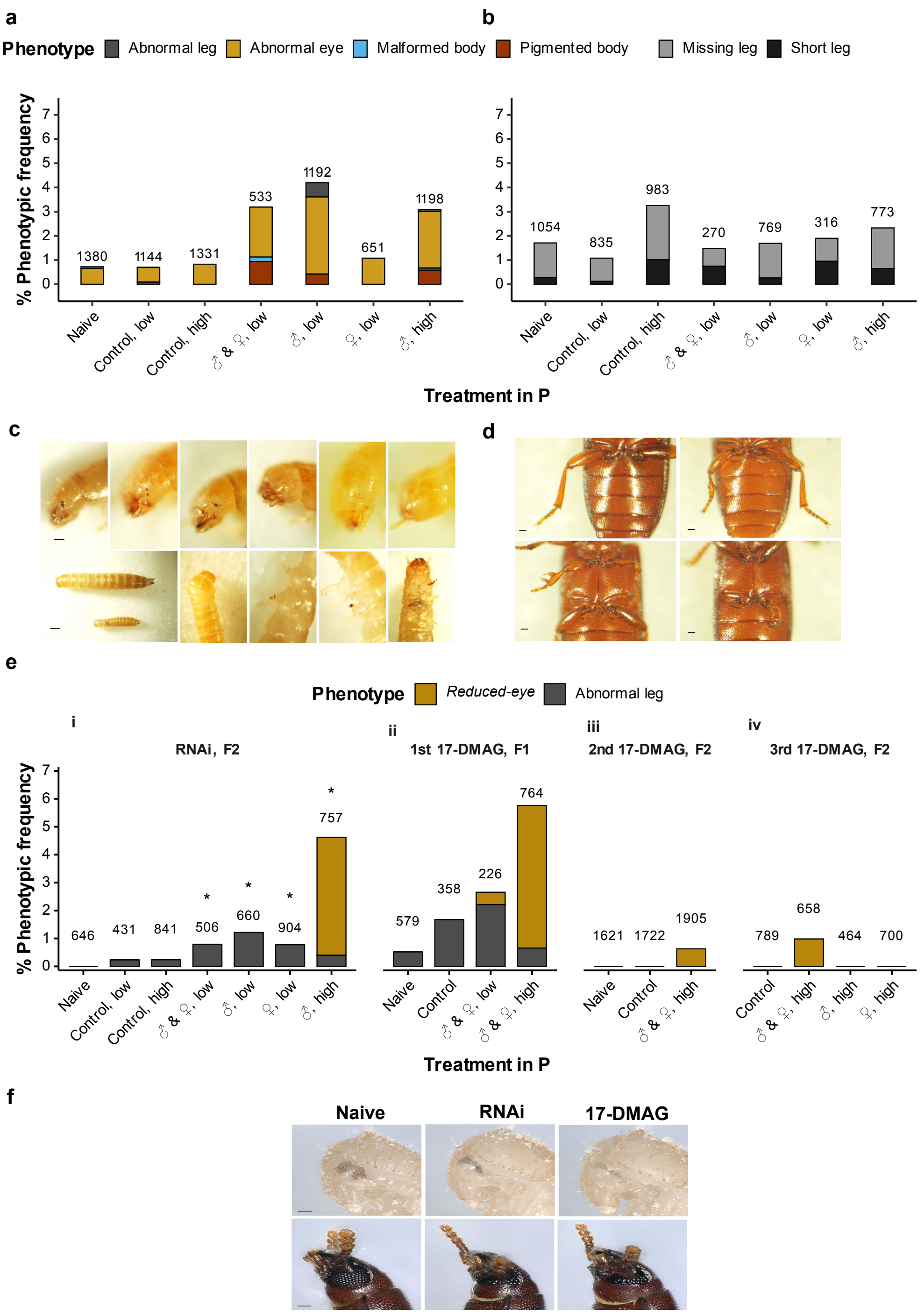

Extended Data Fig. 1 | Release of abnormal phenotypes after HSP90 inhibition via RNAi and 17-DMAG. a, Percentage of abnormal phenotypes in F1-larvae after *Hsp83* knock-down in P via pupal RNAi achieved by injecting pupae with 20 ng/µL (low) or 100 ng/µL (high) dsRNA. Crosses were performed as follows: '♂' indicates the male was treated, '♀' indicates that only the female was treated, and '♂&♀' indicates that both sexes were treated. Control

beetles were injected with *Asna*-dsRNA and crosses were between treated males and females. The total number of individuals screened per treatment (*n*) is shown above each bar. **b,** Abnormal phenotypes in the eclosed F1-adults after *Hsp83* knock-down. **c,** Examples for the abnormal phenotypes in F1-larvae mentioned in (**a**) including abnormalities in larval eyes (distant, scattered, reduced or absent), and other larval body parts (pigmentation, malformed or missing appendages); scale bar: eye (100 µm) and the whole larva (1mm). **d,** Examples for the abnormal phenotypes in F1-adults following parental *Hsp83* RNAi showing short- and missing-legs; scale bars (100 µm). **e**, Release of abnormal phenotype including *reduced-eye* phenotype after HSP90 impairment in P via RNAi or 17-DMAG (performed 3 times). **i**, *Reduced-eye* phenotype in F2 after *Hsp83* knock-down in grandfathers with 100 ng/µL (high) dsRNA. Kruskal-Wallis test: $\chi^2 = 18.25$, df = 6, $p = 0.01$; Wilcoxon *vs.* naïve (♂ & ♀, low: $p_{adj.} = 0.035$), ♂, low: $p_{adj.} = 0.003$), ♀, low: $p_{adj.} = 0.035$), ♂, high: $p = 0.042$). Significant differences are indicated by asterisks *$p < 0.05$, **$p < 0.01$. **ii**, Percentage of abnormal phenotypes in F1 after HSP90 inhibition using 17-DMAG at concentrations of 10 µg/mL (low) and 100 µg/mL (high). Control is food without 17-DMAG. **iii**, A second repeat of 17-DMAG experiment showing the release of *reduced-eye* phenotype in F2. **iv,** A third repeat of 17-DMAG experiment. **f,** Lateral views for *Reduced-eye* phenotype in beetles after RNAi and 17-DMAG treatments (middle and right) compared to the wild-type (left). Two developmental stages are shown: pupae (top), and adults (bottom). Scale bar: 100 µm. Source data are provided via Zenodo and linked in the Data Availability section.

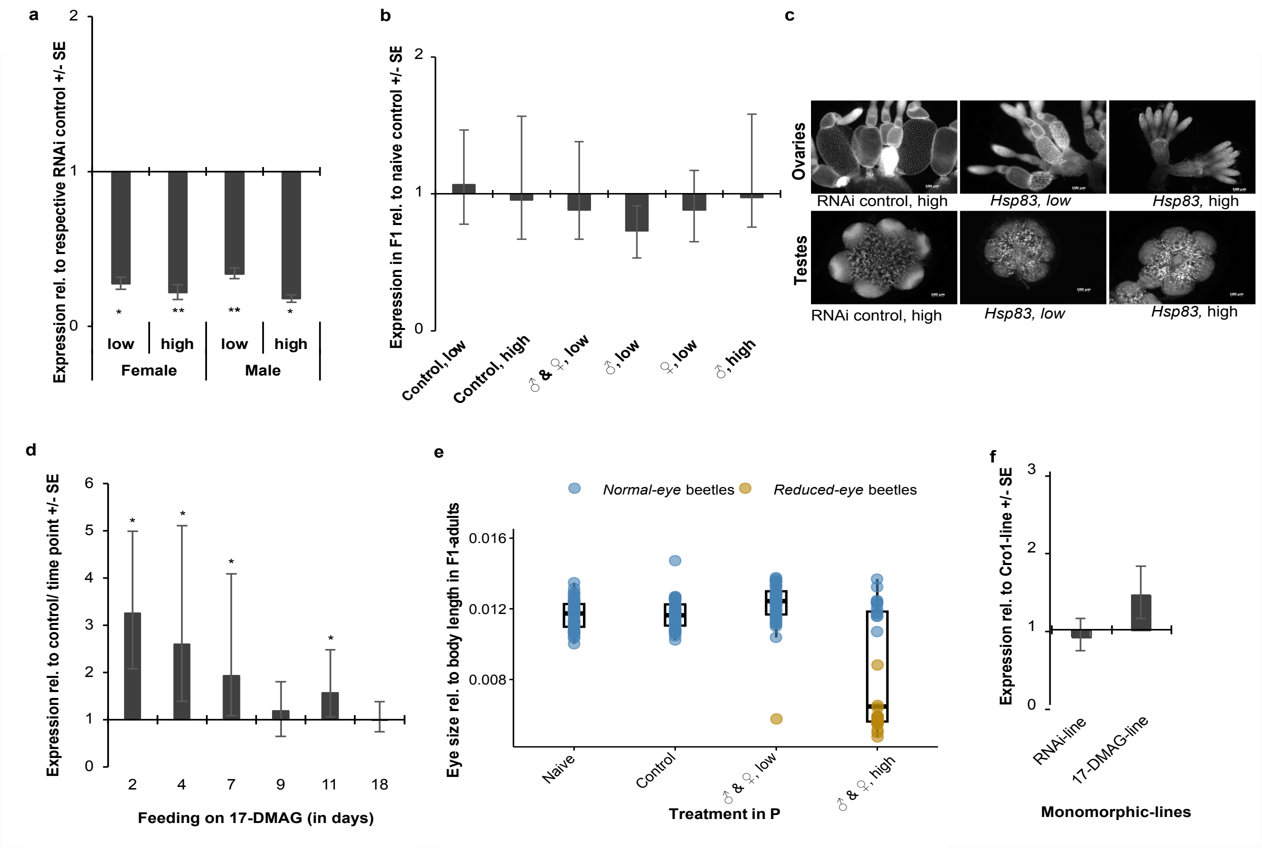

Extended Data Fig.2 | Validation of HSP90 impairment. **a,** *Hsp83* knock-down by RT-qPCR, showing a significant reduction in relative *Hsp83* gene expression in eclosed adult females and males following RNAi-mediated knock-down. Pupae were injected with either 20 ng/µL (low) or 100 ng/µL (high) *Hsp83* dsRNA. Shown is expression ± SEM; n = 4 biological replicate of pools of 5 adults each. REST analysis: Female, low and high: $p = 0.013$ and 0.001, respectively. Male, low and high: $p = 0.005$ and 0.028, respectively. **b,** *Hsp83* expression in untreated F1 larvae (10 days old) produced from *Hsp83*-dsRNA-treated parents, indicating the depletion of ds-RNA. Four biological replicates of pools of 10 larvae each were used. **c,** Gonad phenotypes in eclosed adults after pupal injection with *Hsp83*-dsRNA, showing the drastic effect of *Hsp83* knock-down specially on ovaries. **d,** Expression of *Hsp68a* gene as a molecular marker for the inhibitory effect of 17-DMAG. Expression was measured in larvae fed on flour discs containing 100 µg/mL of 17-DMAG on days 2, 4, 7, 9, and 11, as well as in eclosed adults one week after the last feeding time (day 18). Expression at each time point was compared to the corresponding negative control (flour discs without 17-DMAG). Four biological replicates of pools of 8 individuals each were used. REST analysis: Feeding time points at day 2, 4, 7 and 11: $p = 0.020$, 0.044, 0.028 and 0.030, respectively. **e,** Relative eye size to body length in *reduced-eye* beetles (*F1*) first observed in the initial 17-DMAG experiment, where parental males and females were fed 17-DMAG at 10 µg/mL (low) or 100 µg/mL (high). Each data

point represents an individual with normal eyes (blue dots) or reduced eyes (golden dots). **f,** *Hsp83* relative expression in *reduced-eye* beetles randomly selected from RNAi- or 17-DMAG established monomorphic lines. Three biological replicates of pools of 5 adults each were used. Significant differences indicated by asterisks, $*p < 0.05$, $**p < 0.01$. Source data are provided via Zenodo and linked in the Data Availability section.

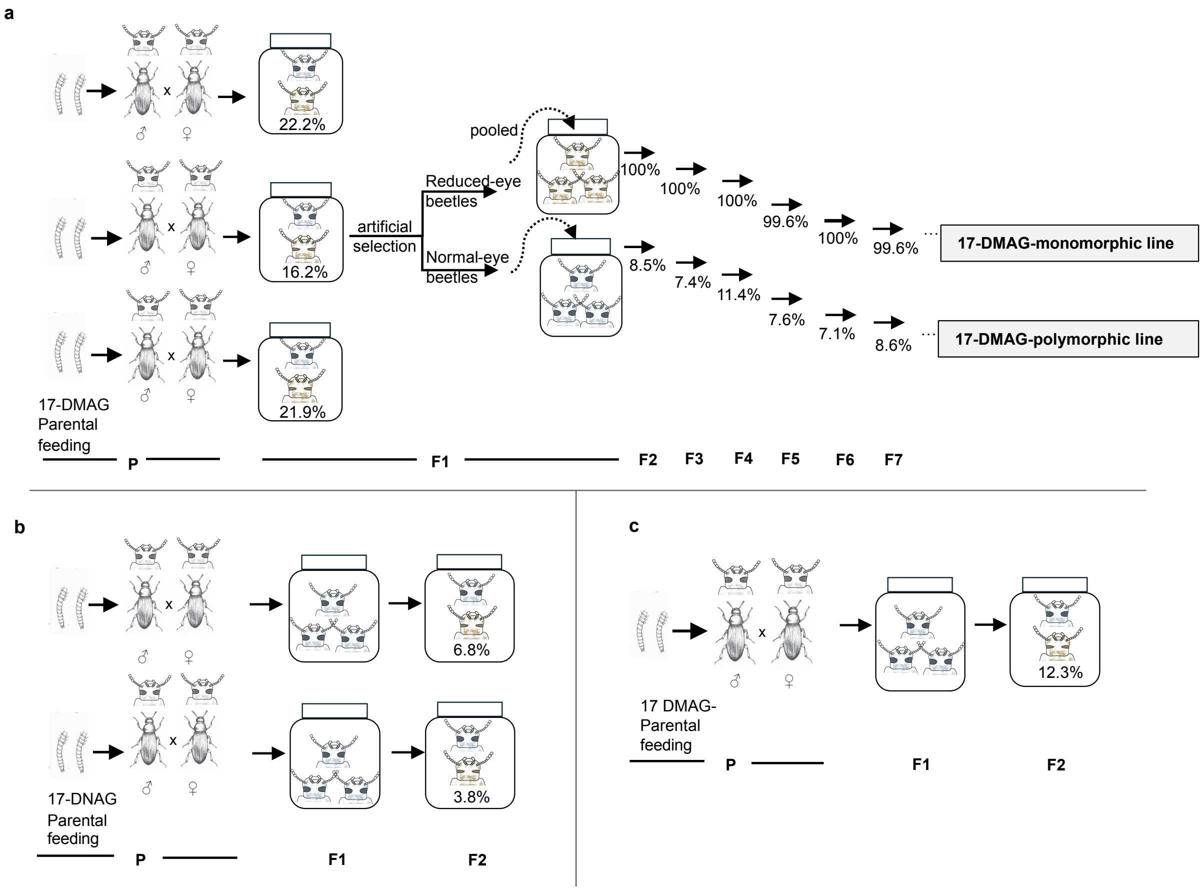

Extended Data Fig. 3 | Inheritance and establishment of *reduced-eye* phenotype following HSP90 inhibition by 17-DMAG. Diagrams illustrating the release and inheritance of the *reduced-eye* phenotype across three independent experiments following chemical inhibition of HSP90 with 17-DMAG. Larvae were fed flour discs containing 17-DMAG (100 µg/mL, high), leading to the emergence of *reduced-eye* beetles in subsequent generations. **a,** First 17-DMAG experiment: A *reduced-eye* phenotype (shown in brown) was observed in F1 offspring from three out of 13 families (percentages indicated on jars). *Normal-eye* phenotype individuals are shown in blue. Mono- and polymorphic lines were established through artificial selection for *reduced-* and *normal-eye* beetles from a single F1 family to control for genetic background. The inheritance of the phenotype was monitored for seven generations. **b**, Second repeat of 17-DMAG experiment: Feeding grandparents with 17-DMAG induced the *reduced-eye* phenotype in F2 offspring. Two F1 pairs out of 17 produced affected progeny. **c**, Third repeat of 17-DMAG experiment: After grandparental feeding with 17-DMAG, the *reduced-eye* phenotype appeared in F2 beetles from one F1 pair out of 15.

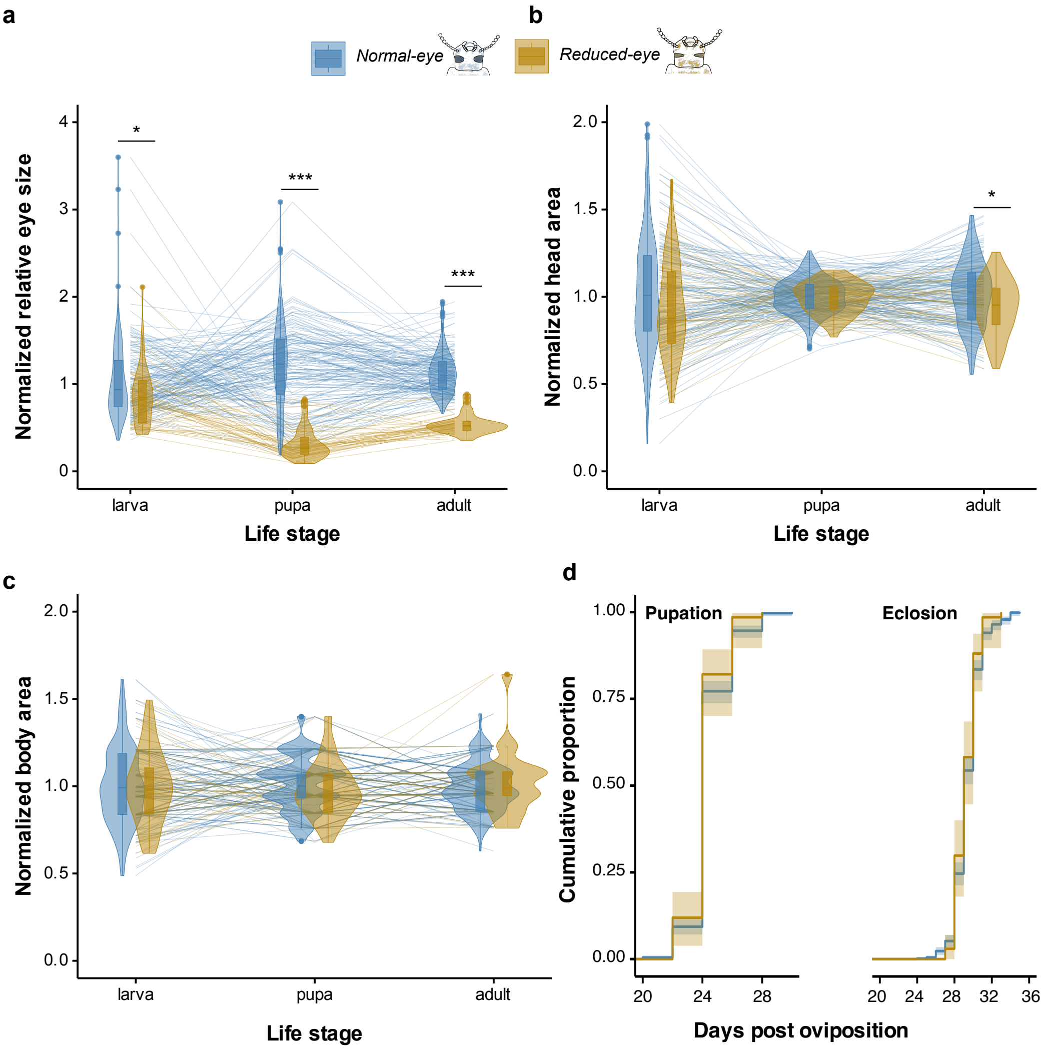

Extended Data Fig. 4 | Quantitative and developmental analysis of the *reduced-eye* phenotype. a, Normalized eye size relative to head area across three developmental stages (larvae, pupae and adults). Two-sided Mann–Whitney U tests were performed: larva: p = 1.60e-2; pupa: p = 9.07e-21 and adult: p = 1.75e-23. **b,** Normalized head area. Two-sided t-test for adults (t= 2.37, df=198, p = 0.0189) **c**, Normalized body area. Panels **a-c**, Lines connect individual beetles tracked from larval to adult stage. Phenotypes (*normal- vs. reduced-eye)* were assigned retrospectively based on adult morphology (n = 156 *normal-eye* and n = 44 *reduced-eye* beetles). **d,** Developmental rate of *reduced-* and *normal-eye* beetles, measured as the day of pupation (left plot) and day of eclosion (adult emergence; right plot). A total of 67 *reduced-eye* and 653 *normal-eye* beetles were examined. Significant differences indicated by asterisks, *p < 0.05, ***p < 0.001. Source data are provided via Zenodo and linked in the Data Availability section.

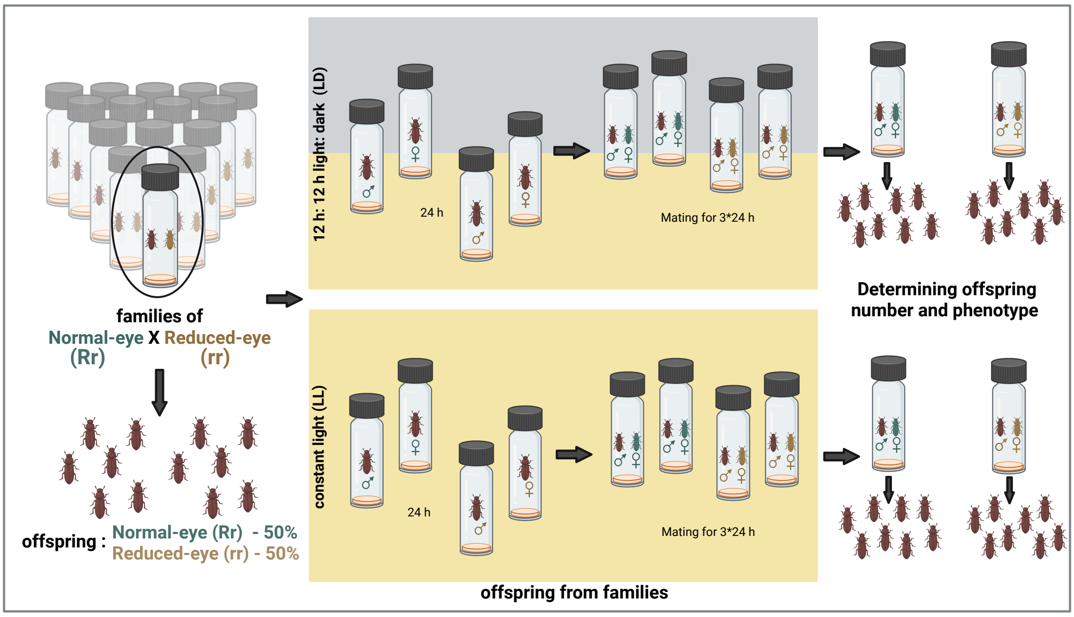

Extended Data Fig. 5 | Diagrams illustrating the establishment of crosses for checking the fitness consequences and penetrance of *reduced-eye* phenotype. Presumed heterozygous *normal-eye* and *reduced-eye* beetles were obtained by backcrossing heterozygous *normal-eye* beetles with monomorphic *reduced-eye* (homozygous) beetles. Beetles with both phenotypes were kept under standard light/dark cycle or continuous light conditions and their offspring number and phenotype were determined. The figure was created in BioRender by R, R. (2025) https://BioRender.com/bql2udu.

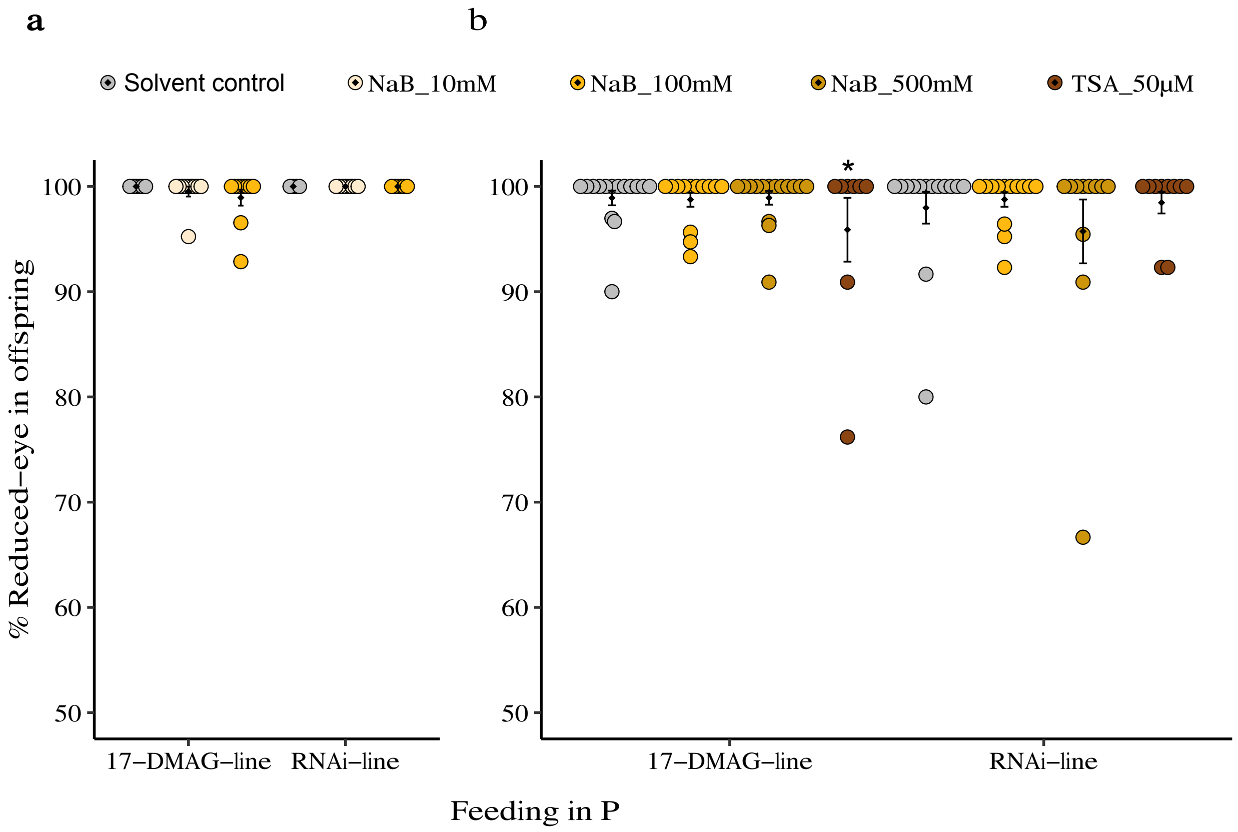

Extended Data Fig. 6 | Penetrance of *reduced-eye* phenotype following HDAC inhibitor treatment. Percentage of affected adult offspring after feeding *reduced-eye* parents +/- standard error of the mean (SEM). **a,** NaB (sodium butyrate) at 10 and 100 mM. (n = 10 families). **b,** NaB, 100 and 500 mM, alongside the HDAC inhibitor TSA (Trichostatin A) at 50 µM. (n = 15 families). Beetles were randomly chosen from monomorphic lines originally established by RNAi- or 17-DMAG-treatment. Each data point represents the *reduced-eye* percent for one single pair. TSA (50µM) in 17-DMAG resulted in a significant 23.8 % reversion (GLM-binomial, z = -2.95, SE = 0.72, $p_{adj.}$ = 0.009). Significant differences are relative to solvent control within each line and indicated by asterisk, *$p < 0.05$. Source data are provided via Zenodo and linked in the Data Availability section.

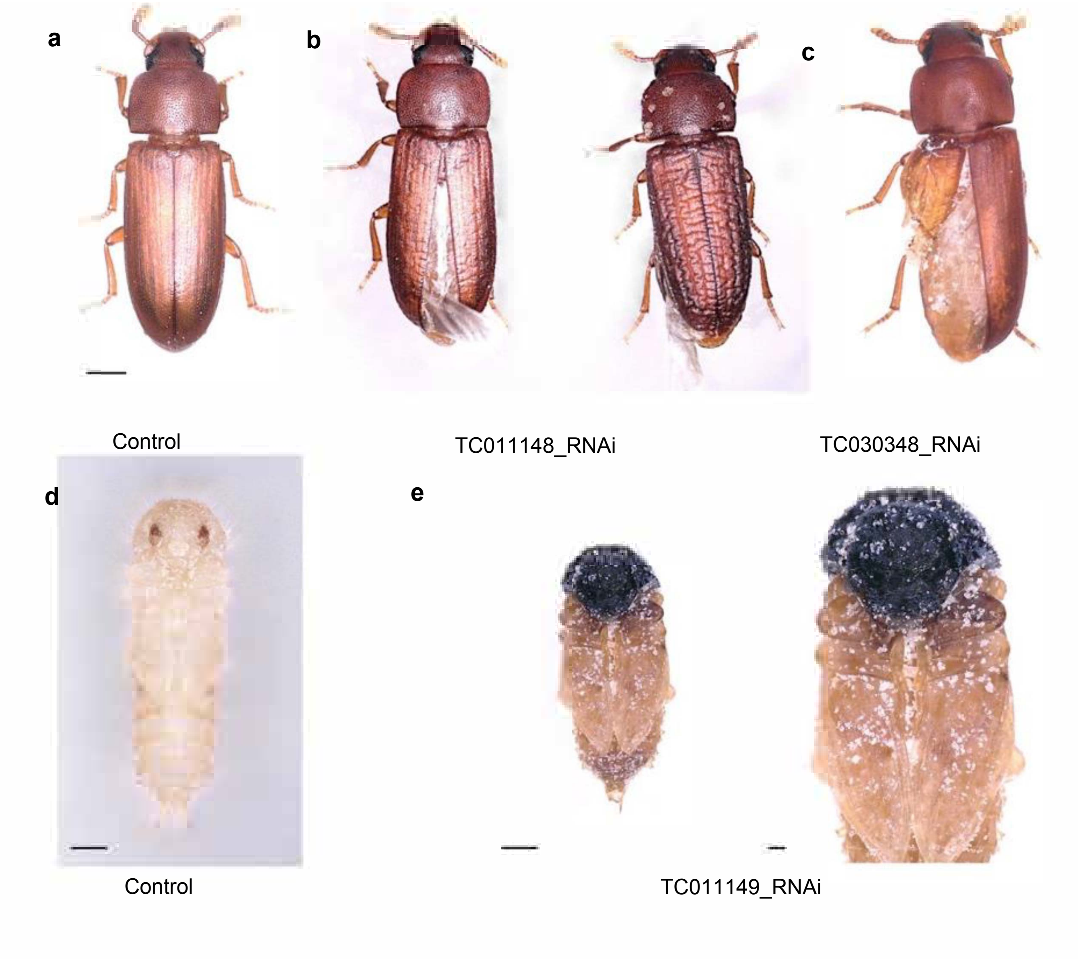

Extended Data Fig. 7 | Abnormal phenotypes in adults after larval and pupal knock-down for candidate genes using RNAi with an injection of 1000 ng/µL. a, Adult control. **b**, Abnormal elytra and chitin structure after knock-down of TC011148 gene. **c,** Abnormal elytra structure in gene knock-downs of TC030348. **d**, Pupa control. **e,** Head regions of dead pupae after TC011149 knock-down. Scale bar 100 µm. Original photos are provided via Zenodo and linked in the Data Availability section.

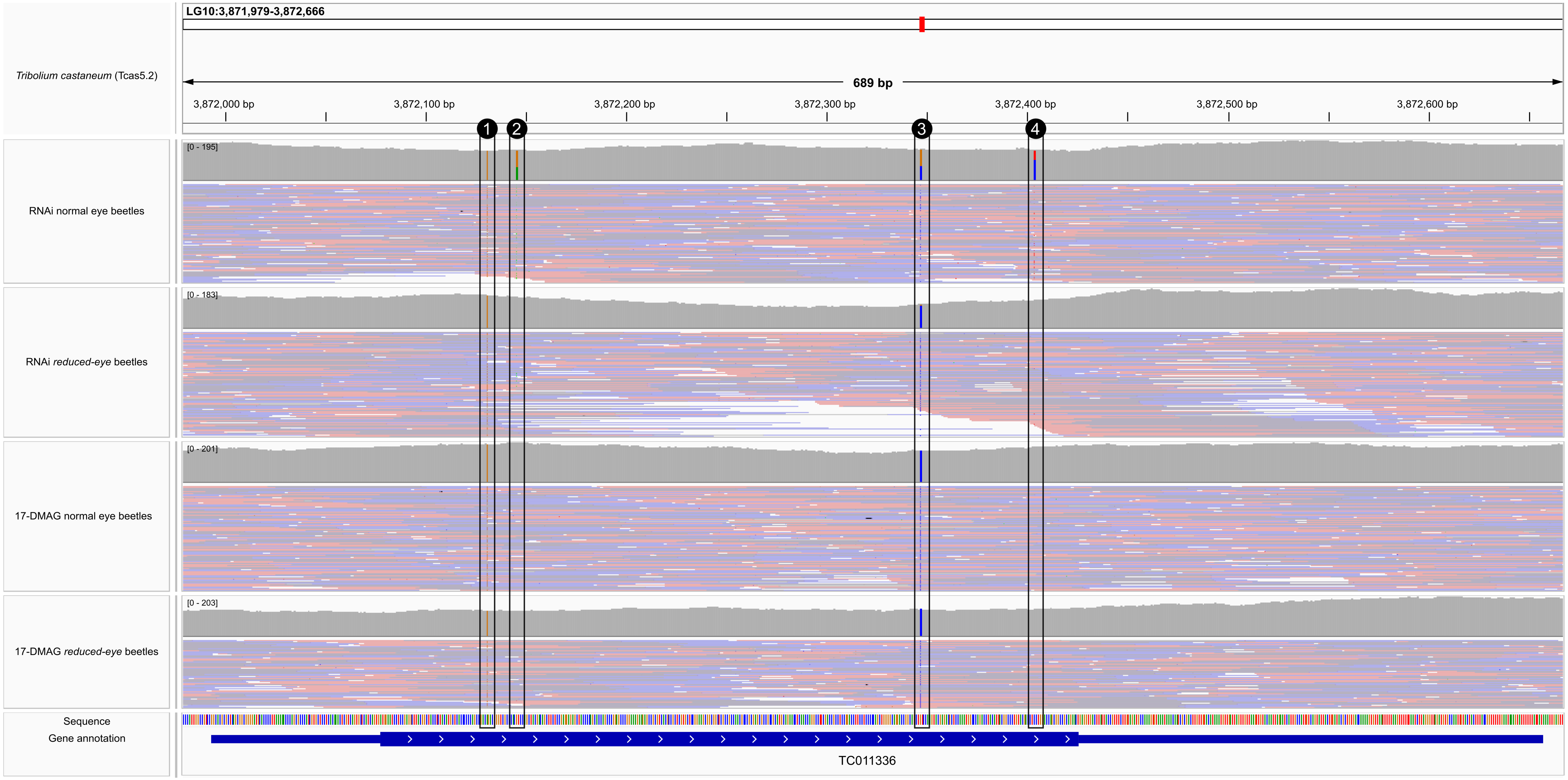
Tribolium castaneum (Tcas5.2)
LG10:3,871,979-3,872,666
689 bp
3,872,000 bp
3,872,100 bp
3,872,200 bp
3,872,300 bp
3,872,400 bp
3,872,500 bp
3,872,600 bp
1
2
3
4
RNAi normal eye beetles
[0 - 195]
RNAi reduced-eye beetles
[0 - 183]
17-DMAG normal eye beetles
[0 - 201]
17-DMAG reduced-eye beetles
[0 - 203]
Sequence
Gene annotation
TC011336

Extended Data Fig. 8 | IGV visualization of alignment files from the four pooled samples analyzed in this study. The first track represents the genomic location of the *ato* gene on LG10 (3,871,936–3,872,660). The second and third tracks show the mapped sequencing data for the *normal-* and *reduced-eye* samples from the RNAi polymorphic line, respectively. The fourth and fifth tracks display the mapped sequencing data for the *normal-* and *reduced-eye* samples from the 17-DMAG polymorphic line. The last track illustrates the *ato* gene model, which contains a single exon. Four synonymous polymorphic mutations (numbered 1 to 4) were identified within the *ato* coding sequence. None of these mutations was consistently associated with the phenotype when comparing *reduced-* and *normal-eye* beetles within each polymorphic line. Mutation 1 at position 3,872,131 (ACA→ACG; Threonine→Threonine) is fixed in both *reduced-* and *normal-eye* beetles across both polymorphic lines. Mutation 2 at position 3,872,146 (ACG→ACA; Threonine→Threonine) is present in *normal-eye* beetles from the RNAi polymorphic line with an allele frequency of ~50 % but is absent in the other samples. Mutation 3 at position 3,872,347 (CTG→CTC; Leucine→Leucine) is present in both *reduced-* and *normal-eye* beetles across both polymorphic lines. Finally, Mutation 4 at position 3,872,404 (CTC→CTT; Leucine→Leucine) has an allele frequency of approximately 30 % in the *normal-eye* sample from the RNAi polymorphic line but is absent in the other samples. The original figure is available on Zenodo https://doi.org/10.5281/zenodo.17120480 (file number 24). Source data are provided in the Data Availability section.

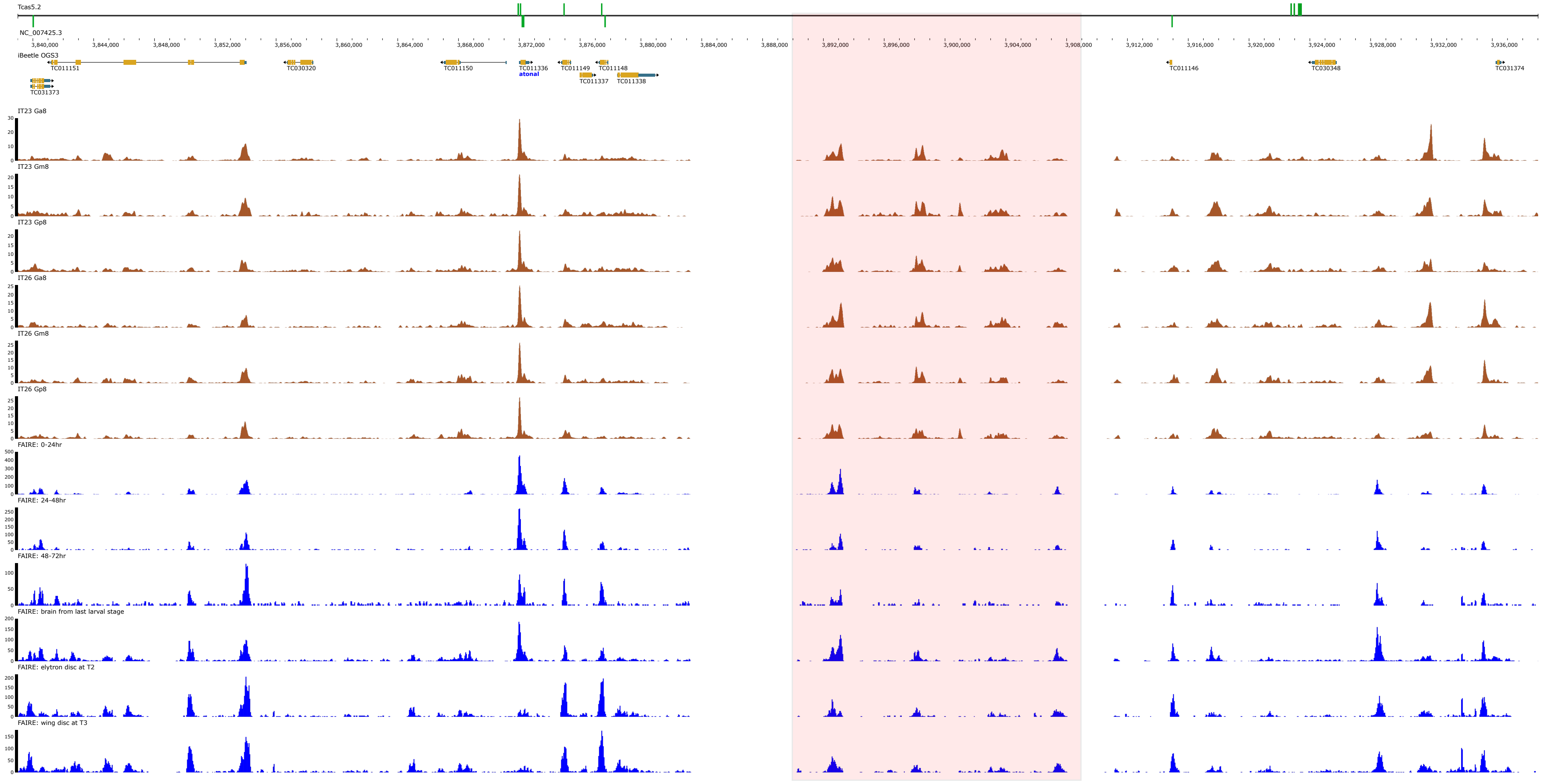
Tcas5.2
NC_007425.3
iBeetle OGS3
TC011151
TC030320
TC011150
TC011336
atonal
TC011149
TC011148
TC011337
TC011338
TC031373
TC011146
TC030348
TC031374
IT23 Ga8
IT23 Gm8
IT23 Gp8
IT26 Ga8
IT26 Gm8
IT26 Gp8
FAIRE: 0-24hr
FAIRE: 24-48hr
FAIRE: 48-72hr
FAIRE: brain from last larval stage
FAIRE: elytron disc at T2
FAIRE: wing disc at T3

Extended Data Fig. 9 | Genome browser view of the 100 kb candidate region showing transcription factor binding motifs (green bars at the top) identified by MEME, overlapping several genes in the region, including *ato*. Brown tracks represent ATAC-seq (Assay for Transposase-Accessible Chromatin) profiles from two developmental time points (23–26 and 26–29 h after egg lay), with three embryo regions (a: anterior, m: middle, p: posterior) per time point[40]. Blue tracks show FAIRE-seq (Formaldehyde-Assisted Isolation of Regulatory Elements) profiles from six samples: three embryonic stages (0–24 h, 24–48 h, and 48–72 h), the second (T2) and third (T3) thoracic epidermal tissues of last instar larvae (containing forewing and hindwing imaginal discs), and brain tissue from last instar larvae[41]. The red-shaded region highlights the overlap between the area lacking annotated genes–yet enriched in highly differentiated SNPs–and peaks of chromatin accessibility in both ATAC-seq and FAIRE-seq datasets, likely corresponding to cis-regulatory elements. The original figure is available on Zenodo https://doi.org/10.5281/zenodo.17120480 (file number 25). Source data are provided in the Data Availability section.

a

Tribolium castaneum Heat shock protein 83 (*Hsp83*), mRNA
NCBI Reference Sequence: NM_001313877.1
20.09.2018

```
ORIGIN
        1 aaaagcaagc gctaagtgaa gagctaagaa gcgacaaatt ttccaagtga taattttccc
       61 aaagcaattt tcaagtgatt tgttcgtgtg tgcattaatt taagcaagat gccggaagaa
      121 aaccaaaatg gagatgtgga aaccttcgcc ttccaggcgg aaatcgccca gttgatgagt
      181 ctgatcatca acaccttcta ctcgaacaag gaaattttcc ttcgggagtt gatttccaat
      241 tcgtcagatg cgttggataa aatccgttac gagtccttga ccaacccttc cagactcgat
      301 tcaggcaaag aactctacat caagatcatc cctaacaaga atgacgggac cttgaccatt
      361 attgacaccg ggatcgggat gactaaagcc gatttggtca ataacttggg caccatcgcc
      421 aagtccggca ccaaagcctt catggaggcc ctccaagctg ggctgacat cagcatgatc
      481 ggtcaattcg gtgttggttt ctactcggct tacttggtag cagacaaggt cacagtcgtt
      541 tcgaagaata acgatgatga gcaatacgtt tgggagtcgt cagctggtgg tagcttcact
      601 gtaacacaag accgtggcga gcctttgggc cgtggcacca agattgtcct tcacatgaaa
      661 gaggaccaaa ccgaattttt ggaagaacac aaaattaaag aaattgtaaa gaaacactcg
      721 cagttcattg gctatcccat caaattggtc gtggagaagg aacgcgagaa ggagttgagc
      781 gacgatgagg ccgaagaaga gaagaaggag gaagaaggcg aagacaagga taaagataag
      841 ccaaagattg aggatgtagg cgaggacgaa gatgaagaca cgaagaagga agataagaaa
      901 aagaagaaga ctattaagga gaaatacaca gaagatgaag aattgaacaa aaccaagccg
      961 atttggacaa gaaacgctga cgatatcagt caggaagaat acggagagtt ttacaaatcg
     1021 ttgactaatg attgggagga ccatttggcc gtcaaacact ttagtgttga gggtcaattg
     1081 gagttccgtg ccctcctctt tgtcccacgt cgcgttccat tcgatctttt cgaaaataag
     1141 aagcgcaaga ataatattaa attatacgtg aggagggttt tcattatgga caactgcgaa
     1201 gaactcatcc ccgaatattt gaactttatc aagggtgtcg tcgattcgga agacttgcct
     1261 ttgaacattt cccgtgagat gttgcaacaa aataagatct tgaaggtcat tcgtaagaat
     1321 ttggtcaaga aatgcctaga gttgttcgag gagttggccg aggataagga cggctacaag
     1381 aaattctacg aacagttctc gaagaatatt aaattgggta ttcatgaaga ctcgcaaaac
     1441 cgggccaaat tgtccgaatt gctccgttat cacacttctg caagtggcga tgaggcttgc
     1501 tctttgaagg actacgtgag ccgcatcaag cctaaccaga aacacattta ttacattact
     1561 ggcgaaagca aggagcaagt ggcgaattcg tcgttcgttg agagggtcaa gaagcgcggt
     1621 ttcgaggtcg tttacatgac tgagcccatt gatgaatacg tcgtacaaca aatgaaagaa
     1681 ttcgacggca aaactctcgt ttcggtcaca aaggaaggtc tcgaattgcc tgaagacgaa
     1741 gaagagaaga agaagcgcga agaagacaaa gccaaatttg aaggactttg caaggttatg
     1801 aagagtatcc tcgataataa ggttgagaag gtcgtggtat cgaaccgtct agtcgaatct
     1861 ccctgctgta ttgttacgtc gcagtatggc tggaccgcca acatggaacg tatcatgaaa
     1921 gcccaagctt tgagagacac ctccactatg ggctacatgg cggccaagaa acacctcgaa
     1981 atcaaccccg accattcaat catcgagaat ttgaggcaga aggctgaggc tgataagaat
     2041 gacaaggctg ttaaagactt ggttattctt ttgtttgaaa ccgctttact cagctctggg
     2101 ttcaccttgg atgagcctca agtccacgca tccaggatct acaggatgat caagctgggt
     2161 ctgggtattg atgaggagga agccatgatc accgaagatg cacaaggagg cgatgcaccc
     2221 tctgctgatg ccgccgagtc cgaggacgcg tcgaggatgg aggaagttga ttaagtgttc
     2281 gaatgttagg acatgtgttc tagtatgttc taattgtcat tcctagtgtt tttttatatt
     2341 tcttaattat ttttataaaa aatgaagtat tattttggcg gcctaccgcc gccgtgttaa
     2401 cacctgtaaa agtctgagtt cgttttttgt gaataaaatt gatttaatta a
//
```

b

Tribolium castaneum *atonal* (LOC659297), mRNA
NCBI Reference Sequence: XM_965616.4
30.11.2024

```
ORIGIN
        1 gtcacactgt attaaaaaac ttcacaacat gcaaaaaaat ttcagtagtt ttttctcctc
       61 atgtatgtaa caaaacggac aaataaaatt tttaagaact gatcaattaa tttggagtca
      121 ccccgcgccc cagttaaccc caatccttcc ctaatctgtc ctcacgtgcc ccccttccac
      181 tggacctacc ctcagcgtcc cattggccgt ccggcacgcg ctcagcggcg gggatgcacc
      241 ccttaaaaac ccagcgccca tcgtgctcct cacaaactcg tcagtgctca tcagtgatgg
      301 aatcctccga ggcttacggc tacctctaca tgctcacccc ccagccgaca gactggtcgc
      361 ccacgtccag ctacgacagc ttctccgagg agagctaccc cagcaccagc ccggagcccg
      421 gaaagaagag ccgcgtcacg ccgctcgtgc tccggaagcg gcggctcgcc gccaacgccc
      481 gcgagcgccg ccgcatgcag aacctcaacc aggccttcga ccgcctccgg accttcctcc
      541 cccagctggg gcaggaccgg cagctgtcca agtacgagac gctccaaatg gcccagacct
      601 acattacggc cctgtacgac ctcctggacc agcggccgca gaactgaggt gacttattac
      661 cgtttcttgt agatgccaaa gtgtaaataa acaatcgcga cttgttgtac ataaatgttg
      721 attagttgtt aagttgtaca attagaatca ttgccattgt cagtattact tgatacgctg
      781 tgaccaaaag tgttaatttt taagtttttt aatcttaggg ttagatgtgc caattgtact
      841 actttaccgg tttttgaata aagtattttt tattataaa
//
```

Extended Data Fig. 10 | A schematic overview of *Hsp83* and *ato* gene mRNA sequence showing the position of the dsRNA-constructs and qPCR primers used in this study. a. *Hsp83* mRNA sequence. Grey highlight indicates the sequence of the ds-RNA construct, with the forward and reverse primers used for RNAi knockdown shown in bold (designed by RS). Forward and reverse primers used for RT-qPCR are also indicated in bold[21]. **b.** *Ato* mRNA sequence. Grey and cyan highlight represents the sequence of 1st and 2nd ds-RNA construct, respectively, provided by Eupheria Biotech under catalog number iB_09565. RT-qPCR primers are shown in bold (designed by ÖS).

Extended Data Tables

Extended Data Table 1 | *Reduced-eye* phenotype after HSP90 inhibition via RNAi and 17-DMAG in P, shown in Fig. 1b. Percentage of *reduced-eye* individuals across affected families following HSP90 inhibition. RNAi and 17-DMAG treatments were applied at different doses. Data include total percentage per treatment, affected families, and the percentage of *reduced-eye* individuals per family. Source data are provided via Zenodo and linked in the Data Availability section.

Group	Treatment	Generation	Total percent of *reduced-eye* per treatment	No. of families with *reduced-eye* out of total	Percentage of *reduced-eye* per family
RNAi	Male, high	F2	4.2	2 out of 15	25.5% (12/47 beetles) and 29.4% (20/68).
1st 17-DMAG	Male & female, low	F1	0.4	1 out of 6	1.4% (1/73)
	Male & female, high	F1	5.1	3 out of 13	22.2% (8/36), 22.0 % (9/41), and 16.6% (22/136)
2nd 17-DMAG	Male & female, high	F2	0.6	2 out of 17	6.8% (6/88) and 3.8% (6/156);
3rd 17-DMAG	Male & female, high	F2	1.0	1 out of 15	12.3% (7/57)

Extended Data Table 2 | Genome-wide analysis of allele frequency differences in *T. castaneum*. Annotated candidate SNPs for the *reduced-eye* phenotype with a shared genetic basis across RNAi and 17-DMAG polymorphic lines (85 SNPs, FDR-corrected $P < 10^{-20}$, LG 10). Statistical analysis was performed using two-sided Fisher's exact test implemented in PoPoolation2 with false discovery rate (FDR) correction for multiple comparisons. DNA was extracted from four pooled samples (n = 50 individuals per pool): one *reduced-eye* and one *normal-eye* pool from each line (RNAi and 17-DMAG). The original table in xlsx format is available on Zenodo https://doi.org/10.5281/zenodo.17120480 (file number 26). Source data are provided in the Data Availability section.

Chromosome	position	–log10 p-values (RNe-vs-RRe)	–log10 p-values (DNe-vs-DRe)	p-values (RNe-vs-RRe)	p-values (DNe-vs-DRe)	–log10 FDR-corrected p-values (RNe-vs-RRe)	–log10 FDR-corrected p-values (DNe-vs-DRe)
LG10	3871149	26.23	27.53	5.89E-27	2.95E-28	21.66	23.08
LG10	3871170	24.80	30.28	1.57E-25	5.25E-31	20.38	25.70
LG10	3873027	24.41	25.00	3.88E-25	9.95E-26	20.02	20.63
LG10	3877148	28.31	30.56	4.84E-29	2.76E-31	23.50	25.97
LG10	3895451	24.79	35.16	1.63E-25	6.93E-36	20.37	30.35
LG10	3895452	25.02	34.82	9.63E-26	1.52E-35	20.58	30.02
LG10	3895540	26.09	34.51	8.07E-27	3.11E-35	21.54	29.72
LG10	3895558	25.80	32.32	1.59E-26	4.76E-33	21.29	27.66
LG10	3895568	27.04	30.52	9.03E-28	3.01E-31	22.40	25.94
LG10	3895579	28.66	28.41	2.19E-29	3.85E-29	23.81	23.91
LG10	3895701	30.04	25.57	9.18E-31	2.67E-26	25.05	21.18
LG10	3895745	32.84	24.54	1.45E-33	2.89E-25	27.49	20.19
LG10	3895760	34.99	32.54	1.02E-35	2.87E-33	29.44	27.86
LG10	3895944	27.83	33.98	1.48E-28	1.04E-34	23.07	29.22
LG10	3896230	26.59	27.04	2.58E-27	9.06E-28	22.00	22.61
LG10	3896390	32.04	32.73	9.04E-33	1.88E-33	26.80	28.02
LG10	3898696	24.47	30.45	3.39E-25	3.53E-31	20.07	25.87
LG10	3898703	26.93	33.00	1.18E-27	9.90E-34	22.30	28.27
LG10	3898952	27.03	42.91	9.27E-28	1.23E-43	22.40	37.71
LG10	3898967	25.85	33.31	1.42E-26	4.90E-34	21.33	28.57
LG10	3899448	27.10	27.13	7.88E-28	7.37E-28	22.45	22.69
LG10	3899464	28.50	32.49	3.18E-29	3.24E-33	23.66	27.82
LG10	3899492	25.70	26.55	2.01E-26	2.84E-27	21.19	22.13
LG10	3899510	25.58	25.71	2.62E-26	1.96E-26	21.09	21.31
LG10	3899718	27.78	37.76	1.65E-28	1.74E-38	23.04	32.84
LG10	3899743	30.38	38.01	4.17E-31	9.84E-39	25.32	33.07
LG10	3899746	28.41	38.67	3.86E-29	2.13E-39	23.59	33.71
LG10	3899759	29.42	31.94	3.82E-30	1.14E-32	24.45	27.29
LG10	3899772	27.19	32.41	6.42E-28	3.92E-33	22.53	27.74
LG10	3899800	24.99	40.31	1.03E-25	4.94E-41	20.56	35.27
LG10	3899812	24.99	43.26	1.03E-25	5.44E-44	20.56	38.05
LG10	3899875	24.79	47.29	1.63E-25	5.13E-48	20.37	41.92
LG10	3899876	25.29	47.18	5.16E-26	6.62E-48	20.82	41.85
LG10	3899884	25.66	49.87	2.21E-26	1.35E-50	21.16	44.18
LG10	3899886	25.94	49.19	1.16E-26	6.51E-50	21.41	43.60
LG10	3899896	27.70	57.32	1.97E-28	4.75E-58	22.97	51.07
LG10	3899906	27.35	58.25	4.51E-28	5.60E-59	22.67	51.52
LG10	3899937	28.19	42.83	6.38E-29	1.49E-43	23.40	37.64
LG10	3899958	27.43	39.58	3.76E-28	2.62E-40	22.74	34.58
LG10	3899959	28.12	40.66	7.54E-29	2.18E-41	23.34	35.58
LG10	3899961	29.88	49.08	1.33E-30	8.29E-50	24.89	43.53
LG10	3899980	25.68	39.85	2.09E-26	1.41E-40	21.18	34.83
LG10	3899996	28.78	47.34	1.66E-29	4.58E-48	23.92	41.95
LG10	3899998	29.08	38.78	8.35E-30	1.67E-39	24.18	33.80
LG10	3900016	31.50	53.99	3.17E-32	1.01E-54	26.32	47.86
LG10	3900022	30.82	41.32	1.50E-31	4.84E-42	25.71	36.20
LG10	3900031	30.12	52.66	7.61E-31	2.21E-53	25.10	46.83
LG10	3900043	29.34	53.53	4.54E-30	2.95E-54	24.39	47.50
LG10	3900050	25.30	45.06	4.99E-26	8.80E-46	20.83	39.79
LG10	3900051	25.43	37.86	3.74E-26	1.38E-38	20.95	32.94
LG10	3900053	25.69	36.45	2.04E-26	3.59E-37	21.19	31.58
LG10	3900069	27.60	52.85	2.52E-28	1.41E-53	22.88	46.90
LG10	3900107	27.36	37.21	4.39E-28	6.15E-38	22.68	32.32
LG10	3900133	26.61	50.06	2.47E-27	8.64E-51	22.01	44.33
LG10	3900136	26.62	36.81	2.39E-27	1.54E-37	22.02	31.93
LG10	3900139	26.74	38.69	1.83E-27	2.03E-39	22.12	33.72
LG10	3900152	27.57	47.21	2.68E-28	6.21E-48	22.86	41.86
LG10	3900200	27.10	49.46	7.98E-28	3.50E-50	22.45	43.81
LG10	3900213	27.80	48.14	1.60E-28	7.28E-49	23.04	42.66
LG10	3900218	28.53	48.35	2.93E-29	4.51E-49	23.69	42.82
LG10	3900225	27.65	32.78	2.22E-28	1.66E-33	22.92	28.07
LG10	3900226	27.45	32.78	3.59E-28	1.66E-33	22.75	28.07
LG10	3900232	30.58	32.57	2.66E-31	2.69E-33	25.50	27.89
LG10	3900244	30.78	47.42	1.64E-31	3.79E-48	25.69	42.01
LG10	3900256	30.09	45.41	8.09E-31	3.88E-46	25.08	40.13
LG10	3900266	27.57	29.29	2.72E-28	5.19E-30	22.86	24.74
LG10	3900267	26.30	29.46	4.97E-27	3.46E-30	21.72	24.92
LG10	3900276	24.68	29.74	2.07E-25	1.84E-30	20.28	25.18
LG10	3900909	25.22	24.43	6.06E-26	3.74E-25	20.76	20.08
LG10	3900914	27.07	25.51	8.42E-28	3.08E-26	22.43	21.12
LG10	3901062	29.53	39.52	2.95E-30	3.04E-40	24.56	34.52
LG10	3901078	26.03	37.73	9.25E-27	1.86E-38	21.48	32.82
LG10	3901087	25.52	28.00	2.99E-26	9.90E-29	21.04	23.53
LG10	3902648	46.82	52.66	1.53E-47	2.17E-53	40.39	46.83
LG10	3902681	30.28	40.61	5.22E-31	2.48E-41	25.25	35.55
LG10	3902775	57.00	30.24	1.00E-57	5.71E-31	50.27	25.67
LG10	3904216	32.57	35.79	2.68E-33	1.62E-36	27.27	30.94
LG10	3904455	31.90	57.45	1.26E-32	3.54E-58	26.69	51.07
LG10	3904549	32.27	42.64	5.36E-33	2.29E-43	27.02	37.47
LG10	3904711	39.06	34.58	8.81E-40	2.64E-35	32.80	29.78
LG10	3904722	38.04	36.81	9.16E-39	1.55E-37	31.91	31.93
LG10	3904728	37.25	37.58	5.57E-38	2.64E-38	31.22	32.67
LG10	3904755	34.44	41.55	3.61E-35	2.80E-42	28.97	36.41
LG10	3904758	35.54	40.41	2.91E-36	3.85E-41	29.85	35.36
LG10	3906982	26.22	38.94	6.05E-27	1.14E-39	21.66	33.96

Extended Data Table 3 | Expansion of candidate region for eye phenotype. Identification of a 100 kb region (3.8 -3.9 kb) overlapping twelve genes on linkage group 10 across RNAi and 17-DMAG polymorphic lines; genes were nominated based on the position of potential SNPs (genes in bold were studied by RNAi). The original table in xlsx format is available on Zenodo https://doi.org/10.5281/zenodo.17120480 (file number 27). Source data are provided in the Data Availability section.

GeneName*	TranscriptId	description	variants_impact_HIGH	variants_impact_LOW	variants_impact_MODERATE	variants_impact_MODIFIER	variants_effect_3_prime_UTR_variant	variants_effect_5_prime_UTR_premature_start_codon_gain_variant	variants_effect_5_prime_UTR_variant	variants_effect_downstream_gene_variant	variants_effect_intron_variant	variants_effect_missense_variant	variants_effect_splice_region_variant	variants_effect_start_lost	variants_effect_stop_gained	variants_effect_stop_lost	variants_effect_synonymous_variant	variants_effect_upstream_gene_variant
TC011146	TC011146_001	uncharacterized	1	1	6	226	0	0	0	96	0	6	1	0	0	1	1	130
**TC011148**	TC011148_001	Adult-specific cuticular protein ACP-20	0	6	3	298	2	0	0	171	7	3	2	0	0	0	5	119
**TC011149**	TC011149_001	Adult-specific cuticular protein ACP-20	0	12	1	370	0	0	0	200	7	1	1	0	0	0	12	163
TC011150	TC011150_001	uncharacterized	0	19	4	551	3	1	4	244	151	4	1	0	0	0	17	149
TC011151	TC011151_001	Nose resistant to fluoxetine protein 6-like Protein [Source:UniProtKB/TrEMBL%3BAcc:D6X413]	0	38	10	1020	1	0	1	135	583	10	4	0	0	0	35	303
**TC011336**	TC011336_001	Atonal [Source:UniProtKB/TrEMBL%3BAcc:D6X416]	0	4	0	391	0	0	0	178	0	0	0	0	0	0	4	213
**TC011337**	TC011337_001	uncharacterized	0	21	21	305	2	0	1	146	11	21	2	0	0	0	19	147
TC011338	TC011338_001	uncharacterized	0	34	19	219	23	0	0	32	3	19	0	0	0	0	34	161
**TC030320**	TC030320_001	Odorant receptor [Source:UniProtKB/TrEMBL%3BAcc:D6X414]	0	42	21	626	0	0	0	351	33	21	5	0	0	0	38	246
**TC030348**	TC030348_001	Odorant receptor [Source:UniProtKB/TrEMBL%3BAcc:D6X424]	0	33	4	496	22	0	0	203	17	4	2	0	0	0	31	256
TC031373	TC031373_001	uncharacterized	0	9	0	363	13	0	3	165	8	0	0	0	0	0	9	174
TC031373	TC031373_002																	
TC031374	TC031374_001	uncharacterized	0	0	0	299	5	0	0	160	0	0	0	0	0	0	0	134

Extended Data Table 4 | Functional analyses of the six candidate genes. Genes were screened for SNP impact, expression profiles, and known roles in developmental processes. Survival and abnormal phenotypes are shown after larval and pupal knock-down using RNAi in beetles from the Cro1 population.

**Gene ID**	**Gene Symbol**	**Gene description**	**Gen Bank**	**RNAi-knockdown effect**							
				**Larval RNAi**				**Pupal RNAi**			
				**1st Block**		**2nd Block**		**1st Block**		**2nd Block**	
				**Survival**	**Phenotype**	**Survival**	**Phenotype**	**Survival**	**Phenotype**	**Survival**	**Phenotype**
TC011148	LOC659500	Adult-specific cuticular protein ACP-20	XM_965802	90%	-Abnormal elytra structure<br>-Abnormal chitin structure	95.6%	-Abnormal chitin structure<br>-Low reproductive success	-	-	-	-
TC011149	LOC659364	Adult-specific cuticular protein ACP-20	XM_965677	92.7%	Normal	100%	Dark head region on dead pupae	-	-	-	-
**TC011336**	**LOC659297**	**Atonal**	**XM_965616**	**94%**	***Reduced-eye* phenotype**	**86.7%**	***Reduced-eye* phenotype**	**95%**	**Normal**	**95%**	**Normal**
TC011337	LOC103314415	Uncharacterized	XM_015983956	80%	Low reproductive success	100%	Low reproductive success	87.5%	Low reproductive success	71.4%	Normal
TC030320	LOC107398753	Odorant receptor Or1-like	XM_015983957	82.5%	Low reproductive success	95.5%	low reproductive success	57.9%	Low reproductive success	77.1%	Low reproductive success
TC030348	LOC107398752	Odorant receptor 43a-like	XM_015983954	90%	Low reproductive success	88.9%	Normal	100%	-Abnormal elytra structure<br>-Normal (yellow eye) offspring<br>-Low reproductive success	92.5%	Low reproductive success

Extended Data Table 5 | Sequencing quality metrics for *normal*- and *reduced-eye* beetles from RNAi and 17-DMAG lines.

Sample Name	% GC	Ins. size	Mean coverage (X)	Mapping rate
**RNAi, *Normal-eye* beetles**	31%	209	355.1	99.60%
**RNAi, *Reduced-eye* beetles**	31%	222	300.7	99.60%
**17-DMAG, *Normal-eye* beetles**	31%	221	360	99.60%
**17-DMAG, *Reduced-eye* beetles**	31%	208	292.2	99.50%

Extended Data Table 6 | Overview of statistical models used in this study. All statistical analyses were performed in R (version2024.04.2+764) using RStudio.

	**Response variable**	**Fixed factors**	**Model used**	**Package**
**Morphological variation**	Abnormal phenotype in percent	Treatment in P (crossings after RNAi or 17-DMAG)	Kruskal-Wallis test	dplyr
**Eye mearuments**	Eye ara / relative eye size / Head area or Body area	Eye Phenotype under each developmental stage	Mann-Whitney U tests or t-tests	dplyr
**Ommatidia number**	Number of ommatidia	Eye phenotype	Two-sample t-test	stats
**Reproductive success as fitness measure**	Number of adult offspring	Light-phenotype combination / Fam. no. as Random factor	GLMM, negative binomial error dis.	lme4
**Development**	Pupation and adult emergence times	Eye phenotype of individuals	Cox proportional hazards model	survival
**Mendel inheritance**	*Reduced-eye* proportion in F2	each Crossing	Chi-square goodness-of-fit test (test Mendel ration)	stats
**Penetrance of phenotype after light stress**	*Reduced-* and *normal-eye* counts in offspring	Light condition	GLMM, bionomial error dis.	lme4
		each light condition	Chi-square goodness-of-fit test	stats
**Trait reversion after HDACs inhibitors**	*Reduced-* and *normal-eye* counts in offspring	HDAC inhibitor concentration for each beetle line	GLM, bionomial distribution	stats
