# Supplementary material for: HSP90 as an evolutionary capacitor drives adaptive eye size reduction via atonal
Source: Nat Commun. 2025 Oct 20;16:9277. doi: 10.1038/s41467-025-65027-0 (PMC12537963; doi:10.1038/s41467-025-65027-0)
Supplement: Supplementary file 3 — Reporting Summary [file 41467_2025_65027_MOESM3_ESM.pdf]

nature portfolio

Corresponding author(s): Joachim Kurtz

Last updated by author(s): Sep 15, 2025

Reporting Summary

Nature Portfolio wishes to improve the reproducibility of the work that we publish. This form provides structure for consistency and transparency in reporting. For further information on Nature Portfolio policies, see our Editorial Policies and the Editorial Policy Checklist.

Statistics

For all statistical analyses, confirm that the following items are present in the figure legend, table legend, main text, or Methods section.

n/a	Confirmed	
☐	☒	The exact sample size (*n*) for each experimental group/condition, given as a discrete number and unit of measurement
☐	☒	A statement on whether measurements were taken from distinct samples or whether the same sample was measured repeatedly
☐	☒	The statistical test(s) used AND whether they are one- or two-sided<br>*Only common tests should be described solely by name; describe more complex techniques in the Methods section.*
☐	☒	A description of all covariates tested
☐	☒	A description of any assumptions or corrections, such as tests of normality and adjustment for multiple comparisons
☐	☒	A full description of the statistical parameters including central tendency (e.g. means) or other basic estimates (e.g. regression coefficient) AND variation (e.g. standard deviation) or associated estimates of uncertainty (e.g. confidence intervals)
☐	☒	For null hypothesis testing, the test statistic (e.g. *F*, *t*, *r*) with confidence intervals, effect sizes, degrees of freedom and *P* value noted<br>*Give P values as exact values whenever suitable.*
☒	☐	For Bayesian analysis, information on the choice of priors and Markov chain Monte Carlo settings
☒	☐	For hierarchical and complex designs, identification of the appropriate level for tests and full reporting of outcomes
☒	☐	Estimates of effect sizes (e.g. Cohen's *d*, Pearson's *r*), indicating how they were calculated

Our web collection on statistics for biologists contains articles on many of the points above.

Software and code

Policy information about availability of computer code

Data collection	No software was used.
Data analysis	1. Statistical Analysis: We utilized R (version 2024.04.2) for all statistical computations, ensuring robust data analysis.<br>2. Quantitative Measurements: ImageJ, an open-source image processing program, was employed for precise quantitative measurements.<br>3. Scale Bar Adjustments: Inkscape, a vector graphics editor, was used to adjust scale bars in images.<br>4. RT-qPCR Analysis: Gene expression data from RT-qPCR experiments were analyzed using REST 2009 software, which employs statistical models to assess up and down-regulation in gene expression studies.<br>4. Genetic Analysis:<br>- Sequencing: Paired-end 150-bp sequencing was conducted on a NextSeq 2000 system at the Core Facility Genomics, University of Münster.<br>- Quality Assessment: Raw read quality was evaluated using FastQC (version 0.11.7).<br>- Read Filtering: Trimmomatic (version 0.38) was employed to remove low-quality reads and adapter sequences.<br>- Alignment: Filtered reads were aligned to the Tribolium castaneum reference genome (Tcas5.2; GenBank accession: GCA_000002335.3) using BWA-MEM (version 0.7.17).<br>- Alignment Quality Evaluation: QualiMap (version 2.2.1) was used to assess mapping quality.<br>- Data Processing: SAMtools (version 1.13) facilitated coordinate sorting of alignment files, while Picard’s MarkDuplicates (version 2.20.0) identified and marked duplicate reads.<br>- SNP Analysis:<br>-- Allele Frequency Comparison: The fisher-test.pl script from PoPoolation2 was utilized to perform Fisher’s exact test on allele frequency differences for each SNP position.<br>-- Visualization: The distribution of –log10 FDR-corrected p-values across the genome was visualized using R (version 4.3.2).

-- Variant Calling: GATK's HaplotypeCaller (version 4.1.2.0) conducted joint-variant calling, with VCFtools (version 0.1.16) employed to exclude indels from the raw call set.
-- SNP Annotation: SnpEff (version 5.2e) provided annotations on the impacts of filtered SNPs on protein-coding genes.
-- Candidate Gene Analysis: Potential candidate genes were examined for associations with high-impact SNPs using the Integrative Genomics Viewer (IGV) and their expression patterns were reviewed via NCBI's Genome Data Viewer.

For manuscripts utilizing custom algorithms or software that are central to the research but not yet described in published literature, software must be made available to editors and reviewers. We strongly encourage code deposition in a community repository (e.g. GitHub). See the Nature Portfolio guidelines for submitting code & software for further information.

Data

Policy information about availability of data

All manuscripts must include a data availability statement. This statement should provide the following information, where applicable:

- Accession codes, unique identifiers, or web links for publicly available datasets
- A description of any restrictions on data availability
- For clinical datasets or third party data, please ensure that the statement adheres to our policy

The Raw data, original photos, plots, stat. analysis and the crossponding R scripts are uplodad on Zenodo. https://doi.org/10.5281/zenodo.17120480
The raw sequencing data generated in this study have been submitted to the NCBI Sequence Read Archive (SRA) under accession number PRJNA1209380.

Research involving human participants, their data, or biological material

Policy information about studies with human participants or human data. See also policy information about sex, gender (identity/presentation), and sexual orientation and race, ethnicity and racism.

Reporting on sex and gender	*Use the terms sex (biological attribute) and gender (shaped by social and cultural circumstances) carefully in order to avoid confusing both terms. Indicate if findings apply to only one sex or gender; describe whether sex and gender were considered in study design; whether sex and/or gender was determined based on self-reporting or assigned and methods used. Provide in the source data disaggregated sex and gender data, where this information has been collected, and if consent has been obtained for sharing of individual-level data; provide overall numbers in this Reporting Summary. Please state if this information has not been collected. Report sex- and gender-based analyses where performed, justify reasons for lack of sex- and gender-based analysis.*
Reporting on race, ethnicity, or other socially relevant groupings	*Please specify the socially constructed or socially relevant categorization variable(s) used in your manuscript and explain why they were used. Please note that such variables should not be used as proxies for other socially constructed/relevant variables (for example, race or ethnicity should not be used as a proxy for socioeconomic status). Provide clear definitions of the relevant terms used, how they were provided (by the participants/respondents, the researchers, or third parties), and the method(s) used to classify people into the different categories (e.g. self-report, census or administrative data, social media data, etc.) Please provide details about how you controlled for confounding variables in your analyses.*
Population characteristics	*Describe the covariate-relevant population characteristics of the human research participants (e.g. age, genotypic information, past and current diagnosis and treatment categories). If you filled out the behavioural & social sciences study design questions and have nothing to add here, write "See above."*
Recruitment	*Describe how participants were recruited. Outline any potential self-selection bias or other biases that may be present and how these are likely to impact results.*
Ethics oversight	*Identify the organization(s) that approved the study protocol.*

Note that full information on the approval of the study protocol must also be provided in the manuscript.

Field-specific reporting

Please select the one below that is the best fit for your research. If you are not sure, read the appropriate sections before making your selection.

☐ Life sciences ☐ Behavioural & social sciences ☒ Ecological, evolutionary & environmental sciences

For a reference copy of the document with all sections, see nature.com/documents/nr-reporting-summary-flat.pdf

Ecological, evolutionary & environmental sciences study design

All studies must disclose on these points even when the disclosure is negative.

Study description

The study investigated the effects of HSP90 inhibition (via 17-DMAG treatment and RNAi knockdown) on Tribolium castaneum, where impairing HSP90 led to a heritable reduced-eye phenotype that increased fitness under continuous light. A factorial design with treated and control groups was used, and genome sequencing linked the phenotype to a specific region, while RNAi experiments identified the atonal gene as responsible. Each individual beetle served as an experimental unit, technical replicates were used for RT-qPCR experiments, and the chemical inhibition experiment was independently repeated to confirm reproducibility. In all experiments, the number of biological replicates was sufficient and determined based on previous studies.

Research sample

In our research, we used the model insect the red flour beetle Tribolium castaneum, Croatia 1 strain (Cro1). This strain was collected from a wild beetle population in Croatia in May 2010, so it represents a genetically diverse population. Stock animals were established from random mating pairs of multiple individuals (165 pairs) and their offspring were used to generate the stock population. The beetles were adapted to laboratory conditions for at least 35 generations prior to the start of our experiments. They were maintained as non-overlapping generations under standard rearing conditions (30°C and 70 % relative humidity with 12 hour light/dark cycle) in heat sterilized (at 75°C) organic wheat flour (Type 550) with 5 % brewer's yeast. The life cycle is about one month under the standard rearing conditions.

Sampling strategy

Beetles were reared in plastic boxes filled with one third 5% yeasted-flour. For our experiments, beetles were randomly picked up from the rearing boxes. The sample size was determined based on previous published literature.

Beetles were randomly assigned to treatment and control groups for all experiments where multiple groups were compared. In cases where random allocation was not feasible, such as selecting reduced-eye beetles for specific analyses, covariates were controlled by using individuals of similar age and sex.

Data collection

1. Morphological Assessment Post-HSP90 Impairment
Procedure: Examined larvae and adult beetles under a dissection microscope for variations in body size, color, head, thorax, abdomen, antennae, eyes, mouthparts, legs, and urogomphi.
Imaging: Captured notable traits by photographing CO_2-anesthetized beetles using a Canon digital camera attached to a Zeiss Axioskop compound microscope.
Data Recording: Conducted by RS.
2. Eye Size, Body, and Head Area Measurements
Procedure: Positioned CO_2-anesthetized beetles ventrally and imaged them with a Keyence digital microscope (VHX-900F).
Analysis: Performed measurements using ImageJ software.
Data Recording: Conducted by ÖS for Mendelian inheritance experiments and Bulk Segregation Analysis.
3. Fitness Experiments
Procedure: Evaluated offspring numbers and adult eye phenotypes using a dissection microscope.
Data Recording: Conducted by RR.
4. Developmental Monitoring
Procedure: Visually monitored beetle development.
Data Recording: Conducted by TP.
5. RT-qPCR Experiments
Sample Selection: Randomly selected beetles from treatment and control groups, when needed.
6. Genetic Analysis of Reduced-Eye Phenotype
Sample Preparation: ÖS extracted DNA samples and constructed sequencing libraries.
Sequencing: Processed libraries on a NextSeq 2000 system at the Core Facility Genomics, University of Münster.
Data Analysis: Bioinformatic processing conducted by ME.

Timing and spatial scale

The sampling time depended mainly on the art of experiment and was determined based on previous published literature.
Spatial scale: Data were collected from laboratory-reared beetles maintained in controlled rearing boxes.

Data exclusions

To ensure robust statistical power, families that were allowed to lay eggs for three days and produced fewer than 10 offspring were excluded from the analysis. Additionally, when the egg-laying period was limited to a single day, offspring counts below five or above 55 were excluded to maintain reliable results. The exact number of exclusions for each treatment is detailed in the statistical analysis section.

Hsp83 knockdown using a high ds-RNA concentration resulted in complete female sterility. However, one out of 30 families produced 16 eggs under this treatment. We excluded this family from the analysis, as it likely indicates that the introduced ds-RNA did not achieve the expected knockdown effect.

For qPCR analyses, each biological replicate was measured in two technical replicates, and the mean Cp value was normally used. If the standard deviation between the two technical replicates exceeded 0.5, this was considered indicative of a pipetting or technical error. In such cases, the replicate causing the high deviation was excluded, and the remaining replicate was used if its Cp value was consistent with the other biological replicates.

Reproducibility

The reduced-eye phenotype observed following HSP90 inhibition with 17-DMAG was validated through two additional independent experiments, consistently yielding reproducible results as depicted in Figure 1b and Extended Data Figure 1e. This phenotype was also observed when HSP90 was impaired by RNA interference, indicating that the reduced-eye phenotype is a consistent outcome of HSP90 inhibition.

Randomization

Beetles were randomly chosen from either the naive CRO1 stocks or the establifhed monomorphic- and polymorphic lines, depending on the aim of the experiment.

	RT-qPCR samples were taken randomly from each treatment.
Blinding	To minimize bias, the majority of morphological assessments were conducted under blinded conditions. However, in experiments need the selection of reduced-eye beetles, blinding was not feasible.

Did the study involve field work? ☐ Yes ☒ No

Reporting for specific materials, systems and methods

We require information from authors about some types of materials, experimental systems and methods used in many studies. Here, indicate whether each material, system or method listed is relevant to your study. If you are not sure if a list item applies to your research, read the appropriate section before selecting a response.

Materials & experimental systems

n/a	Involved in the study
☒	☐ Antibodies
☒	☐ Eukaryotic cell lines
☒	☐ Palaeontology and archaeology
☐	☒ Animals and other organisms
☒	☐ Clinical data
☒	☐ Dual use research of concern
☒	☐ Plants

Methods

n/a	Involved in the study
☒	☐ ChIP-seq
☒	☐ Flow cytometry
☒	☐ MRI-based neuroimaging

Animals and other research organisms

Policy information about studies involving animals; ARRIVE guidelines recommended for reporting animal research, and Sex and Gender in Research

Laboratory animals	In our experiments, we utilized both male and female Tribolium castaneum from the Croatia 1 (Cro1) strain, examining all three developmental stages: larvae, pupae, and adults. T. castaneum, commonly known as the red flour beetle, is a well-established model organism in genetic and developmental studies. The Cro1 strain, specifically collected from Croatia, May 2010: 45° 48′ 55.98, It has been maintained under controlled laboratory conditions to ensure consistency and reliability in research applications.
Wild animals	No wild animals were used in our study.
Reporting on sex	In our study, we utilized both male and female Tribolium castaneum from the Croatia 1 (Cro1) strain, encompassing all three developmental stages: larvae, pupae, and adults. This inclusive approach ensures that our results are applicable across sexes and developmental stages, providing a robust foundation for future research.
Field-collected samples	No field samples were collected in our study.
Ethics oversight	In our study involving Tribolium castaneum, formal ethics approval was not required, as invertebrate research is typically exempt from such oversight. It is not subject to institutional animal care regulations.

Note that full information on the approval of the study protocol must also be provided in the manuscript.

Plants

Seed stocks	*Report on the source of all seed stocks or other plant material used. If applicable, state the seed stock centre and catalogue number. If plant specimens were collected from the field, describe the collection location, date and sampling procedures.*
Novel plant genotypes	*Describe the methods by which all novel plant genotypes were produced. This includes those generated by transgenic approaches, gene editing, chemical/radiation-based mutagenesis and hybridization. For transgenic lines, describe the transformation method, the number of independent lines analyzed and the generation upon which experiments were performed. For gene-edited lines, describe the editor used, the endogenous sequence targeted for editing, the targeting guide RNA sequence (if applicable) and how the editor was applied.*
Authentication	*Describe any authentication procedures for each seed stock used or novel genotype generated. Describe any experiments used to assess the effect of a mutation and, where applicable, how potential secondary effects (e.g. second site T-DNA insertions, mosiacism, off-target gene editing) were examined.*
